# Effect of short-term methylphenidate on social impairment in children with attention deficit/hyperactivity disorder: systematic review

**DOI:** 10.1186/s13034-022-00526-2

**Published:** 2022-11-28

**Authors:** Sarit Alkalay, Orrie Dan

**Affiliations:** Department of Psychology, The Center for Psychobiological Research, Max Stern Jezreel Valley Academic College, P.O.B. 72, 10806 Sede Nahum, Israel

**Keywords:** Attention deficit hyperactivity disorder (ADHD), Methylphenidate, Children, Theory of mind, Non-verbal emotional cues, Social behaviors

## Abstract

Attention Deficit/Hyperactivity disorder (ADHD) is one of the most common disorders in school-age children. In addition to learning difficulties associated with the disorder’s core symptoms of inattention and hyperactivity, children with ADHD display substantial social impairments. Methylphenidate (MPH) in formulations such as Ritalin or Concerta mitigates inattention and hyperactivity, but the effects of the therapy on social behavior in children with ADHD are not clear. This review aims to determine the effectiveness of short term (up to 6 months) MPH treatment on three domains of social skills in children aged 6–14 with ADHD: (i) Recognition of nonverbal emotional expressions, which are a marker of inherent (unlearned) social understanding, (ii) theory of mind (ToM) components that relate to learned cognition and social communication, and (iii) social competence in everyday environments. 15 relevant studies were identified based on inclusion/exclusion criteria. The results show mixed effects: the overall social performance as evaluated by parents, teachers or peers, and some components of ToM, were found to improve following a weeks-long course of MPH treatment. However, the effects of the medication are less clear when evaluating momentary/nonverbal social responses such as reactions to emotional facial expressions. While the findings of this review indicate that an MPH medication regime of order weeks to months could improve, to a degree, social impairment in children with ADHD, more studies are required to identify the medications’ mechanism and confirm such a conclusion.

## Introduction

Attention-Deficit/Hyperactivity disorder (ADHD) affects an estimated 2–12% of school-age children worldwide (see, for example, [1–7]). Increasing awareness has led to the diagnosis of many children as early as pre-school or the first years in school, namely ages 4–8 (see, for example, [8]).

The disorder is defined by a range of inattention and/or hyperactivity–impulsivity symptoms [9, 10] that cause impairments in cognitive domains (see, for example, [10–13]). Somewhat less discussed, but also prevalent in individuals with ADHD are deficiencies in social competences, namely, social behavior, social cognition, and social outcomes [14–30]. For example, children with ADHD display a tendency to interrupt others, fail to follow instructions, and exhibit aggression and rule breaking [31–36]. The result in some cases is rejection by their peers [26, 37–41] and/or poor friendship quality [37–40], which may lead to lifetime-long maladjustment (see, for example, [42]).

Methylphenidate (MPH) in formulations such as Ritalin^©^ or Concerta^©^ has been found to be highly effective in the treatment of ADHD symptoms, alone or in combination with behavioral therapy, (see, for example, [43, 44]). As a result, MPH is recommended as the first line of treatment for ADHD and is the most broadly prescribed ADHD medication for children and adolescents [45–49]. MPH has been shown to improve the performance of children with ADHD on measures of attention, impulsivity and memory, as well as reduce omission errors (namely, no response to target stimuli, linked to inattention) and commission errors (response to non-target stimuli, associated with impulsivity) (see, for example, [50–52]).

Appropriate social functioning is a complex decision-making process based on the understanding of emotional and motivational states in others, the application of prior learning of social norms to the choice of behavior, and the ability to act appropriately [53]. As a result, social skills require both long-term learning (i.e., knowledge of acceptable social behaviors) and ‘in the moment’ components (i.e., acquisition of situational inputs and enacting appropriate responses) [53]. In ADHD, it is suggested that acquisition is intact (i.e., children with ADHD possess knowledge about “what to do”), but performance and fluency are impaired (they “don’t do what they know”) [46, 54–56]. Performance issues are related to misunderstanding and mis-interpretation of emotional cues or emotional facial expressions (see, for example, [57–59], while fluency is demonstrated by weak social problem-solving skills [33, 60–62]. It is expected that MPH would improve momentary processes in children with ADHD, namely, the encoding of emotional inputs or the ability to control actions. Indeed, reports by parents and teachers suggest that children treated with MPH show improvement in social interactions and a reduction in oppositional or aggressive behavior at home and in the classroom [51]. However, it is unclear whether MPH treatment also affects long-term acquisition of social behaviors, and thus the overall social performance of children with ADHD.

The aim of this review is to examine the effects of MPH on specific components of social performance in children 6–14 years old with ADHD. We chose this age range based on several considerations. First, diagnosis of preschoolers with ADHD is complicated due to other aspects of their development (see, for example, [63]). As a result, younger children are more likely to be diagnosed with ADHD during their early school years- namely around age 6 (see, for example, [64–66]). This is also enhanced by the transition of children from the family as their core social environment to a peer-based focused environment which starts when they enter school at age 6, when deficits in the social domain become more apparent (see, for example, [67]). Once diagnosed, treatment is likely to follow age-specific treatment guidelines as set by the American Academy of Child and Adolescent Psychiatry [68, 69] and the American Academy of Pediatrics [AAP] [70] that recommend for preschool-age children diagnosed with ADHD parent- or teacher- administered behavior therapy as the first-line treatment. However, for children 6 years of age and older, the AAP recommends prescribing medication and/or behavior therapy, with a preference for both treatments in combination [68–70].

Our cutoff at the older age range of our sample is 11–13, namely, pre-adolescence [71]. ADHD changes with age, especially in the adolescent years (see, for example, [72–74]): the major physical changes in adolescence affect emotions, cognition, and social interactions [75] which are at the heart of ADHD manifestation and our study. To avoid a possible obscuring effect due to those hormones-related changes, we did not include studies with participants older than 14.

Three domains of social skills are examined, as representatives of the different steps in the execution of social performance:

- Recognition of emotional inputs, as measured through accurate identification of emotions: communication of emotions is a core, essential step in social functioning. Individuals that are impaired in their ability to recognize emotions in others cannot perform socially in a competent manner [20, 23, 30]. Some processes of emotion communication, such as facial expressions, have been shown to be fundamental and universal [76–88], implying that humans are biologically “hard-wired” to recognize them. Tests present simple emotional cues: pictures of emotional facial expressions, postures, gestures, or other paralanguage cues. Thus, these tests are largely independent of cognitive assessment or prior learning, and evaluation of the effect of MPH on performance yields information on the ‘in the moment’ processes that do not rely on experiences.
- ‘Theory of mind’ (ToM): ToM is a components of social cognition that relates to the skills required to manage social communication and relationships [89]. In typically developing children, ToM is attained by age 3–4 through social interactions that promote conceptualization of the abstract and subjective nature of mental processes and psychological causality [89]. The result is the ability to note motives, beliefs, and feelings in others. However, gaining and refining ToM continues throughout the school years and into adulthood [89]. ToM development depends on memory systems (e.g., short- and long- term declarative memory, emotional memory), on language, and on executive functions. As a result, tests of the effect of MPH on ToM in children with ADHD examines the accumulation of social and emotional concepts.
- Social competence (as assessed by self, peers, family and/or teacher evaluations): social competence is generally defined as the effective functioning of individuals within social context, and may be divided into three aspects: attainment of society-defined accomplishments, global (external) judgment of social competences, and evaluation of peer acceptance [90]. Assessment of social competence therefore reveals the long-term experience of the individual and their perception. Laboratory experiments evaluate the temporary effect of MPH on a specific social function. However, using evaluation of social competence through self, peer, parental or teacher evaluations provides understanding of the effect of the medication on the child’s actual, everyday performance, encompassing short-term and long-term aspects.

Better understanding of the effects of MPH on these three domains will enable answering questions important for the treatment of children with ADHD, such as: does medication with MPH improve social deficits in children with ADHD? If it does, are the effects short term and ‘in the moment’, or long term? Answers might help design appropriate medication regimes to address social deficits in children with ADHD.

## Methods

We follow a framework adapted from Whittemore and Knafl [91] for the conduction of integrated reviews, and relevant aspects of the PRISMA 2020 [91]. The process is divided into several stages that include problem identification, search of the literature, data extraction and evaluation, data analysis and presentation of the results.

### Data sources and searches

The search included papers published between 1980 and 2021 in APA PsycNet, MEDLINE, PubMed, ISI Web of Science, Scopus.

### Study selection

Studies were eligible for the review if they were peer reviewed and published in the English language. Inclusion criteria were based on the following Boolean descriptors: (ADHD OR “attention deficit disorder” OR “attention deficit hyperactivity disorder” OR “attention-deficit/hyperactivity disorder”) AND (social) AND (methylphenidate OR MPH or stimulant) AND (children OR youth). Exclusion criteria included:

- non-reviewed publications (e.g., conference papers, MS or PhD thesis), papers published in languages other than English, or review papers.
- studies in which the average age of the participants was not in the target range of 6–14 years.
- case studies (namely, studies with a small sample size of less than 6 participants).
- studies where the participants were not consistently diagnosed with ADHD based on DSM III [93], DSM IV [94] or DSM V [9].
- studies where a substantial fraction (40% or more) of the ADHD group had additional psychological disorders, namely, comorbidities, such as oppositional defiant disorder (ODD), conduct disorder (CD), or learning disability (LD).
- studies where the ADHD group was also medicated with drugs other than MPH.
- studies whose focus was on comparing different formulations or dosages of MPH.
- studies where the effects of medication were evaluated after more than 12 months of a medication regime: Social cognition in children changes relatively rapidly, so that such long-term studies may include effects not directly related to the medication.

### Screening and data extraction

The abstracts were assessed by the authors to evaluate compliance with the inclusion/exclusion criteria. Papers that complied with the criteria were then read in total. The authors extracted the relevant data including testing methodology, tested traits/behaviors, and outcomes.

## Results

### Study selection and characteristics

**Fig. 1:**
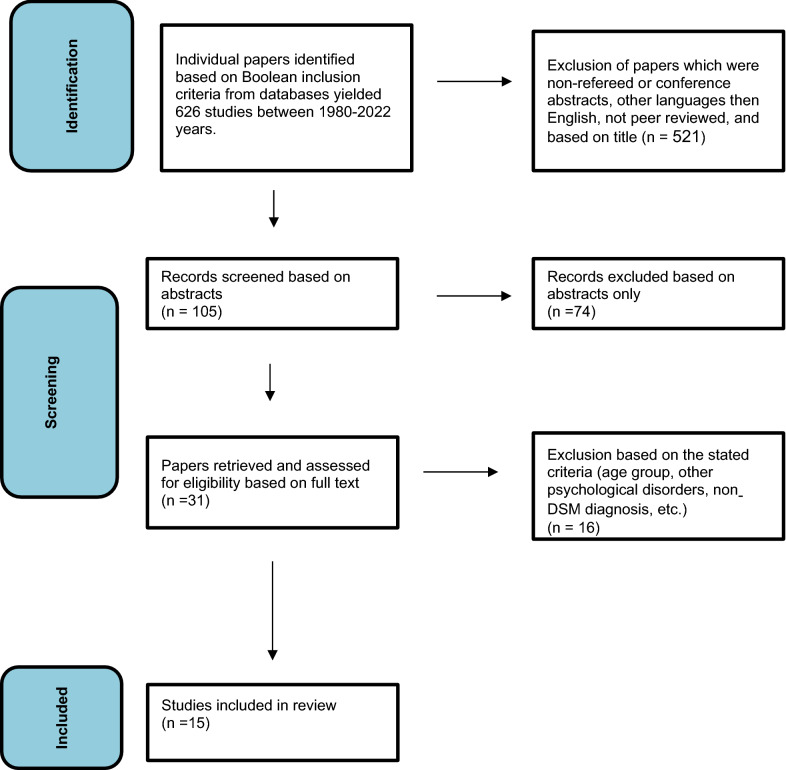
The review process flow diagram

Database Boolean search results yielded 626 papers. These were examined individually by the authors to exclude those that did not comply with the exclusion criteria listed above. The process is described in Fig. 1.

Table 1 lists the 15 papers that satisfied the criteria for this review, and the basic study parameters: size of the study (defined by the number of participants with ADHD), age range, and gender distribution. Also noted is the distribution of ADHD sub type (when specified): (I)- predominantly inattentive type, (H)- predominantly hyperactive/impulsive type, (C)- combined type. Tests conducted include: reading the Mind in the Eyes Test (RMET); Benton Face Recognition Test (BFRT); Empathizing Quotient (EQ); Suspiciousness Rating Scale (SRS); Bryant Index of Empathy (BEI); Griffith Empathy Measurement (GEM); Empathy Response Task (ERT); Diagnostic Analysis of Nonverbal Accuracy-2 (DANVA-2); Social Perception Behavior Rating Scale (SPBRS); Life Participation Scale (LPS); Interpersonal reactivity index (IRI); “faux-pas” recognition task (FPR); Social Adjustment Inventory for Children and Adolescents (SAICA); Event Related Potential (ERP).

**Table 1:** Demographic and study data for the reviewed papers

1st Author	Year	Age range (Average ± SD)	Number ADHD group^a^	Male (%)	Subtype^b^	Groups and control	Dependent variable
Abikoff [95, 96]	2004	7.0–9.9 (8.2 ± 0.8)	34	93%	Not noted	3 experimental, no control	Social functioning -Parent, child, and teacher ratings
Bottelier [97]	2017	10–12	35	100%	60%I, 40%C	1 experimental, 1 control	Amygdala reactivity
Demirci [17]	2016	8–15 (10.8 ± 1.5)	38^3^	48%^e^	37%I, 28%H, 35%C^e^	3 experimental, 1 control	Face and emotion recognition (RMET; BFRT)
Golubchik [98]	2019	7–17 (10.8 ± 2.7)	25	84%	Not noted	1 experimental, No control	Face and emotion recognition (RMET)
Golubchik [99]	2017	8–18	26^f^	Not specified	Not noted	1 experimental, No control	Empathy (EQ)
Golubchik [100]	2018	8–18 (12.9 ± 2.5)	30^f^	58%	Not noted	1 experimental, 1 control	Suspiciousness (SRS)
Gumustas [101]	2017	8–14 (10.86 ± 1.85)	65	82%	Not noted	1 experimental, 1 control	Empathy and emotion expression recognition (BEI; GEM; ERT; DANVA-2)
Hall [102]	1999	7–10	15	80%^c^	77% C, 8.5% H	2 experimental, 1 control	Emotion expression recognition and social perception (DANVA;SPBRS)
Kim [103]	2015	6–18 (9.4 ± 2.4)	116	86%	33.6%I, 5.2%H, 36.2%C^d^	1 experimental, No control	Self control (LPS)
Kobayashi [104]	2020	8–10 (9.8 ± 1.3)	19	95%	21% I, 79% C	1 experimental, No control	Response to facial expressions measured by oxyhemoglobin responses in the right inferior occipital region
Levi-Shachar [105]	2020	6–12 (9.4 ± 1.6)	50	56%	Not noted	1 experimental, 1 control	Social cognition and Oxytocin level
Maoz [106]	2014	6–12	24	67%	54% I, 46% C	1 experimental, No control	Interpersonal Reactivity Index, Theory of Mind and Empathy (IRI; FPR; computerized ToM task)
Maoz [107]	2019	(10.28 ± 1.64)	24	66%	Not noted	1 experimental, 1 control	Theory of Mind and Empathy (IRI; FPR)
Shang [108]	2020	7–16 (9.61 ± 2.41)	168/83 mph	87%	Not noted	2 experimental	Social adjustment, Social Adjustment Inventory for Children and Adolescents (SAICA)
Williams [109]	2008	8–17 (13.79 ± 2.33)	51	100%	Not noted	1 experimental, 1 control	Response to facial expressions (ERPs)

^a^This number represents only the children in the ADHD group. Some studies also included a group of ADHD + a specific comorbidity (e.g., ODD), but these were not taken into account and are not presented in this table

^b^(I): predominantly inattentive type, (H): predominantly hyperactive/impulsive type, (C): combined type

^c^Of the entire study, namely, ADHD + Typically Developing (TD) groups

^d^25% not specified/determined

^e^The total number of ADHD children was 60; However, only 38 of them received MPH, while the others received atomoxetine. Statistics are for the entire ADHD group

^f^The study also included a group of children with ADHD and ODD, which we did not include in our analysis

### Effect of MPH on recognition of emotional inputs

The Diagnostic Analysis of Nonverbal Accuracy (DANVA) was used by Hall et al. [102] to examine the response of children with ADHD to images of adults and children showing various emotions though facial expression, gestures, or paralanguage cues. The ADHD participants were previously diagnosed with ADHD and were receiving MPH for treatment of the disorder at the time of the study. They were tested twice: once while medicated and once after a ‘washout’ period of at least 24 h. Their results were also compared to those of matched group of typically-developing (TD) children acting as control. No significant differences were found between the TD control group and the ADHD group either when medicated with MPH or when un-medicated, in all aspects of the DANVA evaluation (facial expression, gestures, or paralanguage) [102] (It should be noted that the study also included a group of children with ADHD and LD, but the results of this group are outside this review’s parameters).

Kobayashi et al. [104] investigated the hemodynamic response in the temporal and occipital regions of children with ADHD when presented with angry or happy facial expressions. All children were regularly medicated with MPH prior to the study. A double-blind test was conducted in two sessions that followed a washout period of 4 days. In each session, children underwent a baseline, medication-free test, followed by a post-medication test using wither MPH or a placebo. The researchers [104] found that MPH did not affect the response to happy facial expressions, which involved a significant increase in the oxygenated hemodynamic (oxy-Hb) in the right inferior occipital area. In contrast, the response to angry expressions showed a significant increase after MPH administration in the left inferior occipital area when compared to the medication free or placebo.

In a functional Magnetic Resonance Imaging (fMRI) study, Bottelier et al. [97] examined the activity in the amygdala in drug-naïve children with ADHD that were presented with angry or fearful faces (as well as neutral images). The children were tested before and after the administration of a short-acting MPH dose and compared to TD children. The researchers [97] found that MPH did not affect the response in the left amygdala but did reduce the response in the right amygdala. In both, the TD response was much higher, so that in regard to the right amygdala, MPH increased the difference between ADHD and TD groups. However, the researchers noted that amygdala reactivity did not correlate with emotional dysregulation as measured with clinical rating scales [97].

A somewhat different methodology was used by Williams et al. [109] and Gumustas et al. [101], where the effect of MPH on the recognition of emotional facial expressions in children with ADHD was examined. Tests were conducted at baseline, before the start of MPH treatment, and again after a period of MPH administration (4 weeks [109] or 12 weeks [101]). The majority of the children with ADHD in these studies were drug naïve, and the others underwent a ‘washout’ period before the baseline testing. In addition, the ADHD group’s results were compared to those of a matched TD control.

Gumustas et al. [101] found that the accuracy of emotional facial expressions was lower in the ADHD group at baseline (pre-treatment) when compared to the TD group. However, the differences were not statistically significant. After 12 weeks of MPH treatment, errors in the recognition of happy and fearful expressions remained the same, but accuracy in identification of sad and angry faces increased but not in a statistically significant manner. Williams et al. [109] found that the accuracy of facial emotion recognition pre-treatment in the children with ADHD was the same for neutral, happy and sad expressions. It was statistically significantly lower, however, for angry and fearful expressions. Following 4 weeks of MPH treatment (and performance of the test while medicated), the children with ADHD showed a significant improvement in their ability to identify fear and anger. However, their performance for these expressions remained impaired when compared to the TD controls [109].

### Theory of mind (ToM) studies

The ‘reading the mind in the eyes’ test (RMET) examines ToM by using photos of the eye region, where participant are asked to identify the emotion portrayed. Unlike the facial expression pictures (e.g., in DANVA) where emotions are clearly defined, RMET illustrates complex mental states such as ‘‘embarrassed’’ and require application of ToM concepts of inferences.

Golubchik et al. [98] tested the performance of children with ADHD in the children’s version of RMET, before and after receiving a dose of MPH. The study confirmed that RMET assesses ToM in the ADHD group by correlating it to social functioning as evaluated by the SDQ screening questionnaire that investigates emotional symptoms, conduct problems, hyperactivity/inattention, peer relationship problems and prosocial behavior. Baseline (un-medicated) testing showed an effect of age but not ADHD severity as evaluated by ADHD rating scale (ADHD-RS) score. No significant change in the RMET score was found between the baseline/un-medicated and medicated with MPH tests. It should be noted that the previous medication history of the ADHD group was not clearly stated.

Demirci et al. [17] tested the long term effect of MPH on children with ADHD using RMET. The baseline, medication-free RMET found that the ADHD group had a significantly higher number of errors than a matched TD control group, with the H subtype performing worse than the I subtype. A 3 month long MPH medication regime led to improvement in performance (namely, reduction in the number of errors) in the ADHD group.

Other aspects of ToM were examined by Maoz et al. who used a ‘faux pas’ (FPR) [106, 107] test to measure the ability of children with ADHD to recognize social situations in which a speaker says something without understanding that it could be misinterpreted. They also used a computerized task (TCT) [106] to test the children’s ability to judge mental states using verbal cues and eye gaze. The children performed the tasks in two sessions: One while medication-free after a washout period of at least 24 h, and one after receiving a dose of long- acting MPH. The researchers found that MPH administration led to a significant improvement in performance on all tests. Interestingly, children with poorer baseline ToM performance showed greater improvement when medicated by MPH [106, 107].

More recently, Levi-Shachar et al. [105] examined the effect of MPH vs. placebo on ToM in children with ADHD using vignettes, stories, and drawings that assess emotion recognition, first-order belief and false belief, and advanced components (e.g., humor), as well as an FPR test. Children who were on MPH regularly underwent a washout period of 48 h, and then tested, once when medicated with MPH, and once when taking a placebo. They found that children with ADHD performed worse than TD controls on the ToM test and FPR when un-medicated (namely, medicated with a placebo). However, the differences between the two groups disappeared when the children with ADHD were medicated with MPH.

A related concept to ToM is empathy, which is defined as an affective response based on an assessment of another person’s emotional state. This definition encompasses the cognitive awareness of the inner state of another person—that is, the emotions, thoughts, perspectives, and intentions of the other [101]. Gumustas et al. [101] tested empathy in children with ADHD, using the Bryant Index for Empathy (BEI), which evaluates trait empathy, and the Empathy Response Task (ERT) which evaluates state empathy. The ERT requires verbal responses to fictitious episodes presented as a short narrative about a child and illustrated with a picture where facial features were blank. Responses were scored by computing a match score and an interpretation score. It was found that there were no statistically significant differences between groups (TD, pre-treatment and after 12 weeks of MPH medication) in terms of trait and state empathy levels, although the medication regime did lead to a significant increase in the interpretation sub scores of empathy [101].

### Effect of MPH on (overall) social competencies

A number of studies followed the effect of MPH on measures of overall social performance in children with ADHD, using self, peer, parental or teacher evaluations.

Shang et al. [108] examined the effect of MPH on children with ADHD using self and parent ratings through the Social Adjustment Inventory for Children and Adolescents (SAICA). The test was administered at baseline and periodically following the start of MPH treatment. Parental reports showed consistent and statistically significant improvement after 8 weeks of MPH in both school functions and peer relationships. This improvement continued into week 16, after which it seemed to stabilize. Child self-ratings showed similar trends, although the improvement trend was weaker. Interestingly, neither self nor parental reports observed significant improvement in home and family behaviors.

Golubchik et al. assessed the effect of a 12 week long MPH treatment regime on the Emotional Quotient (EQ) [99] and on suspiciousness and social-function related items such as feeling teased, blaming others, feeling others are out to get you and feeling suspicious of others [100]. As expected, the treatment was shown to reduce the ADHD-RS scores significantly, indicating a reduction in the severity of ADHD symptoms [99]. The MPH regimen was found to increase the Emotional Quotient (EQ) score in the children, indicating an improvement in empathy, with changes in the EQ score significantly correlated with the changes in ADHD-RS [99]. Also, a small but statistically significant reduction in the measures of suspiciousness was observed [100].

Somewhat similar findings after a 12 week long MPH regimen applied to mostly drug-naïve children were observed by Kim et al. [103]. Results from the Life Participation Scale (LPS) showed some improvement in the social/happy scales between baseline and post MPH treatment, although the difference was statistically minor. Analysis suggested that there is a degree of association between the medication-induced reduction in ADHD symptoms and an improved adaptive functioning. It should be noted that although approximately 30% of the children had comorbidities (depression, ODD, tic or anxiety disorder), the relationship between changes in the related comorbidity symptoms showed only a weak to moderate degree of association when compared with the association between ADHD core symptoms and functioning.

Longer-term effects of MPH on children with ADHD were studied by Abikoff et al. [95, 96], who compared the baseline, medication-free tests to 6, 12, 18, and 24 months post treatment onset. Here we focus on the 6 month results only, since as noted above, ageing by more than this period can have significant effects on children. The study used the self and parent Social Skills Rating Scale (SSRS) which measures social skills problem behaviors and academic competence, as well as teacher evaluations through the Taxonomy of Problematic Social Situations (TOPS), which yield a total score as well as scores for six scales: peer group entry, response to peer provocations, response to failure, response to success, social expectations, and teacher expectations. In addition, the researchers conducted direct observations of the children in the school environment. After 6 months of MPH treatment, the self, parent, and teacher ratings showed significant improvement in the children’s performance, as did the observational data.

Overall social skills can also be tested in laboratory settings. Pelham et al. [110] examined the effect of medication (MPH vs. placebo) and expectation of medication using a peer-interaction task that encompassed a range of social skills: perception and interpretation of cues from the other, information integration, choice and enactment of a response. The study compared pre-task self-evaluation to performance as judged by self and by a tester. They found that medication expectancy did not affect children’s performance. Moreover, children that received the MPH did not differ from those that received placebo, as evaluated both by self-report or by a research observer. In contrast, social success, or failure (which were manipulated by the researcher) had a significant effect on self-assessment, regardless of medication status.

## Discussion

ADHD has been linked to social impairment in children [14, 15, 17, 20, 22–25, 27–30]. Stimulants, and in particular MPH, improve cognitive and scholastic deficits in children with ADHD [43, 44]. The aim of this review was to examine how MPH may affect components of social deficits in children with ADHD. To that end, we divided the studies into three categories: the effect of MPH on recognition of emotional inputs, which relates to short-term effects (i.e., ‘in the moment’ performance), on theory of mind (ToM), and on overall social skills, both of which relate to long-term effects.

Core emotional facial expressions such as happy or angry are a universal method of emotion communication that is largely independent of social learning [76–88]. Communication of emotions via nonverbal cues may be more complex, but is also a relatively universal skill (see, for example, [111]) that is closely linked to attention [112]. Studies to date suggest that children with ADHD experience some impairment in the recognition of emotional facial expressions [25, 113], although broadly differing results vary as to the specific emotions that present challenges. Since MPH is known to increase sustained attention in children with ADHD (see, for example, [114–116]), it is reasonable to assume that children with ADHD would perform better on emotion recognition tasks while medicated with MPH. On the other hand, it is unclear whether long-term medication status (namely, children who have been medicated with MPH over a period of time) would have an effect in a similar way.

The results of studies examining the effect of MPH on emotion recognition in children with ADHD are somewhat contradictory. Both Gumustas et al. [101] and Hall et al. [102] did not find significant differences between a TD control group and children with ADHD that were tested while unmedicated, in contradiction of previous findings that show impairments [25, 113]. Thus, it is possible that their study design was not sensitive to the relevant deficits that are characteristic in children with ADHD, and so their additional observation that MPH (either after a 12 week-long medication regime [101] or while conducting the test [102]) had no statistically significant effect on performance is inconclusive. The results of Williams et al. [109] may be more illuminating: at baseline, namely pre-treatment with MPH and medication-free during testing, they found that the accuracy of facial emotion in the ADHD group was poorer for some expressions (angry and fearful) but not for others. MPH significantly improved the children’s performance [109]. However, the second set of tests was conducted after 4 weeks of treatment and while medicated, it is not possible to conclude whether the effect of MPH was due to long-term effects, or to the ‘in the moment’ increased attention imparted by the medication.

The answer to this question is partially given in studies of brain activity: Bottelier et al. [97] used fMRI to examine the response to angry or fearful expressions by drug-naïve children with ADHD, while medication free and while medicated with MPH. Their finding that the medication affected the response in the right amygdala [97] must be related to the medication’s ‘in the moment’ effect, since the children were not on a long-term medication regime. In the Kobayashi et al. [104] study, the children were on a long-term medication regime, and their response recorded while medication-free after a washout period, medicated with MPH or with a placebo. Their finding that there was a more pronounced response to angry (but not to happy) expression while medicated with MPH when compared to the baseline washout test or the placebo, is also an indicator of an ‘in the moment’ effect. However, it is difficult to correlate these findings regarding brain activity to attention or accuracy of emotion recognition. Thus, more studies that clearly distinguish between the effects of long-term and short-term use of MPH are needed.

The next type of studies examined aspects of ToM. Meta-analysis shows that children with ADHD have impaired ToM when compared to their TD counterparts [60]. As noted above, ToM is typically developed by age 3–4 [89], well below the age at which most children are diagnosed with ADHD [48, 49]. However, it continues to evolve in older children, and is related to executive functions [117]. The effect of MPH on ToM in children with ADHD could be associated with its long-term effects on executive functions and enhanced learning but could also improve ‘in the moment’ performance by enhancing attention during testing.

Regarding the short-term effect of MPH on ToM, Golubchik et al. [98] did not find significant change in RMET score between un-medicated and medicated testing of children with ADHD. In contrast, Maoz et al. [106, 107] found MPH enhanced the performance of children when compared to testing while un-medicated. Similarly, Levi-Shachar et al. [105] found that children with ADHD performed worse than TD controls when un-medicated, but matched the TD group when medicated with MPH. Differences in the effect of weeks-long MPH medication regime on ToM are also observed: Demirci et al. found a clear improvement after a 3 months long medication regime [17], but Gumustas et al. did not see any deficits before starting the medication process (when compared to TD children) or improvement after 12 weeks of MPH [101].

Most evaluations of how MPH affects global aspects of social performance in children with ADHD focused on long-term medication regimes. Whether through self, parental or teacher assessments, it is agreed that medication with MPH for a period of 8 weeks or more improves social skills in children with ADHD [95, 96, 99, 100, 103, 104]. In contrast, laboratory testing did not find a significant difference between performance while medicated with MPH or a placebo [110].

Social performance in children is a multi-facet and complex process. For example, Dodge’s Social Information Processing (SIP) proposes six steps [53] that include encoding of cues, interpretation and mental representation of the cues, goal clarification, construction of possible responses, response evaluation and decision, and behavioral enactment. Clearly, some of them rely on momentary abilities (encoding of cues, enactment of behavior) while others rely on acquired learning of social behaviors. The studies reviewed here demonstrate that overall social performance, and some components of ToM, are improved by a weeks-long regim of MPH treatment (reported effects after only 3 months in some studies), and also after medication during the testing situations only. However, despite the expectation that the medication’s improvement in aspects of attention would lead to better performance ‘in the moment’ (i.e., facial recognition), those effects are less clear. It may be cautiously concluded, therefore, that an MPH medication regime continued over time would improve social impairment in children with ADHD. However, more studies are required to confirm this conclusion and to clarify what is the duration of medication needed for observing effects on the social components of facial recognition, ToM and social performance.

## Data Availability

All data and materials are listed in the article.
